# Has Vaccination Anything to Do with the Degeneration of the Human Teeth?

**Published:** 1881-06

**Authors:** F. Richardson


### ARTICLE V.

### *Has Vaccination Anything to do With the Degeneration of the Human Teeth?*

(Paper read at the Midland Counties Branch of the British Dental Association.)

BY F. RICHARDSON, L. D. S.

"Has vaccination anything to do with the degeneration of human teeth?" is a question which, if once raised, ought not to be permitted to rest until it has been conclusively answered, either in the affirmative or negative. To us, as dentists, the subject possesses so much of novelty, that beyond a pamphlet published a few years ago by Mr. Albert Carter, it has not, so far as I am aware, attracted even the passing attention of any member of our profession. And this is the more remarkable, because were the subject of vaccination now brought forward for the first time, we may feel very certain that it would not be allowed to become the accomplished fact we find it at this day, until its possible influence upon the teeth had been thoroughly investigated.

Nearly one hundred years ago, Jenner's famous theory was first announced to the world. In those days both dentists and doctors were almost equally in the dark respecting the origin of the teeth. It is only within the last fifty years that their histology and development have been satisfactorily investigated, and now there are few portions of the human frame, better understood than the dental organs.

Assuming then that this question has never been entertained, and seeing, moreover, that the knowledge and information now within our reach is so far in advance of that which existed at the close of the last century, it is surely incumbent upon us as a body to take up the subject as it was left a hundred years ago, and investigate it under the more enlightened conditions of the present day.

In considering this subject, there are three well-known facts that require to be kept in view, namely, that the teeth are dermal appendages, that their first germs are discernable at a very early period of fœtal existence, and that in its primitive stage each tooth is in the condition of a soft, impressible mass.

At birth, and for some time after, nature is more busily engaged in developing the different organs and building up the frame-work of our complex bodies, than at any subsequent period. And surely if there is a time when she requires to be left to her own devices, it is this; and the most obvious mode of conforming to her requirements is to interfere with her as little as possible, and to keep the infant in as perfect a state of repose as its age and surrounding circumstances will admit of. This, I think, must be self-evident and conclusive; yet what do we find? Why, that at this most critical period of its existence, the law steps in and decrees that before this child, fresh from the hand of its Maker, is fit to take its place in the world, it shall first be subjected to an operation, the effect of which is to throw the whole system into a state of turmoil and confusion, occasionally terminating in death.

The virus or poison thus introduced into the system (whereby an implied omission on the part of the Creator is satisfactorily rectified,) develops into the well-known vaccinia or cow-pock, which, with measles, scarlet fever, small-pox, etc., forms a class of skin affections belonging to the zymotic or blood-poisoning group, the specific action of which consists in producing a change in the blood analagous to fermentation.

That certain of the exanthemata should exert a baneful effect upon the teeth is not to be wondered at, considering the close relationship existing between the latter and the skin. Thus we find that one of the sequelæ of scarlatina consists in the exfoliation of portions of the alveolar border, containing, it may be, some of the developing permanent teeth; and to measles is in part accredited the occurrence of honeycombed teeth, where we find both enamel and dentine more or less disorganized, and consequently more susceptible to adverse influences than those which are more perfectly developed. Externally the crown is more or less distorted from its normal shape, the enamel is deficient both in quantity and quality (indicating an arrest of development and nutrition,) is frequently pitted and often porous and fragile in character. Internally, we find interglobular spaces, while the tubuli are irregular both in size and arrangement.

Now, seeing that two of this class of disorders are capable of producing such results, surely there can be no reason why another so closely allied as vaccinia should not at least be suspected of a similar evil influence; and I venture to think that the following case which occurred recently in my own practice, points unmistakably to this and no other conclusion.

A few months ago, a lady called upon me with four of her children, whose teeth she wished me to examine. Three of them may be dismissed with the remark that they all had been vaccinated, and in all the permanent teeth were badly honeycombed. In the case of the fourth (a boy,) a very different state of things existed. Here it was the temporary teeth that were honeycombed, which I now exhibit; the permanent set, so far as they were developed, being perfection itself. On making a few inquiries of the mother, she informed me that some months before the child was born, she, when under the influence of one of the usual periodical scares, allowed herself to be revaccinated. Hence, on the assumption that the operation was the cause of the honeycombing of the child's temporary teeth, it would appear that special inflammations may occur during the period of the inter uterine existence. After birth, the operation of vaccination was three times attempted upon the child, but as the mother said, "it would not take."

There are a few facts connected with this case which, I think, deserve careful consideration.

1. Three of the children had been vaccinated in the usual manner, and all had honeycombed teeth.

2. The fourth was not vaccinated, and his permanent teeth were perfect.

3. The mother had been vaccinated when the child was in *utero*. The child's temporary teeth were extensively honeycombed.

4. Vaccination, although three times attempted upon this child, failed each time, presumably because the influence of that which he received through his mother was still sufficient to render the operation abortive.

I therefore can come to no other conclusion than this, namely: that the vaccination performed upon the mother previous to the birth of her child was the direct cause of its temporary teeth being honeycombed. And if this is the correct explanation, and indirect vaccination can so clearly assert itself, surely its influence would not be less potent when exerted after birth.

I venture to think this case points to but one conclusion, namely: that the deformity was the direct result of vaccination.

Time and the limits of my paper will not permit more than a statement of these facts, and I prefer leaving to better hands than mine the discussion which I think they deserve.

With regard to vaccination itself, and looking at it from a purely professional point of view, I venture to think it is an operatien that out of regard to its possible influence upon the teeth, may well be postponed till later in life. Even assuming that it is a protection against small pox, surely all other considerations ought not to be ignored for the sake of this one. Beside, the danger of a child, surrounded by proper sanitary conditions, being attacked by small pox, is surely a very remote contingency, whereas vaccination is direct and immediate. For it seems impossible that the number of infants who suffer from measles, or other serious ailments, before they are three months old (when the vaccination laws first assert their authority over them,) is such as would account for even a fraction of the cases of honeycombed teeth which constantly fall under our notice. In many instances, we are told, either that the child never had a day's illness beyond what is usual after vaccination, or else that he was attacked at an age when we know that even the six-year-old molar is too far developed to exhibit any external traces of adverse influences.

Let us, then, as dentists, acting in the interests of our patients, look well into this matter, so that when the whole subject of vaccination comes on for discussion "in another place" (as there is every probability that it will) we, as a body, may this time have something to say about it, and perhaps our voice may be the means of deciding whether for the future our babies are to be "fermented" according to act of Parliament, or whether they are to remain unleavened as God sent them.—*Monthly Review of Dental Surgery.*

---

